# Articulation Speaks to Executive Function: An Investigation in 4- to 6-Year-Olds

**DOI:** 10.3389/fpsyg.2018.00172

**Published:** 2018-02-26

**Authors:** Nicole Netelenbos, Robbin L. Gibb, Fangfang Li, Claudia L. R. Gonzalez

**Affiliations:** ^1^The Brain in Action Laboratory, University of Lethbridge, Lethbridge, AB, Canada; ^2^Canadian Centre for Behavioural Neuroscience, University of Lethbridge, Lethbridge, AB, Canada; ^3^Department of Psychology, University of Lethbridge, Lethbridge, AB, Canada

**Keywords:** speech articulation, executive function, BRIEF, fricative production, cognition, child development, language development

## Abstract

Executive function (EF) and language learning play a prominent role in early childhood development. Empirical research continues to point to a concurrent relation between these two faculties. What has been given little attention, however, is the association between EF and speech articulation abilities in children. This study investigated this relation in children aged 4–6 years. Significant correlations indicated that children with better EF [via parental report of the Behavior Rating Inventory of Executive Function (BRIEF) inventory] exhibited stronger speech sound production abilities in the articulation of the “s” and “sh” sounds. Furthermore, regression analyses revealed that the Global Executive Composite (GEC) of EF as measured by the BRIEF, served as a predictor for speech sound proficiency and that speech sound proficiency served as a predictor for the GEC. Together, these results demonstrate the imbricated nature of EF and speech sound production while bearing theoretical and practical implications. From a theoretical standpoint, the close link between EF and speech articulation may indicate a common ontogenetic pathway. From a practical perspective, the results suggest that children with speech difficulties could be at higher risk for EF deficits.

## Introduction

Two components play a fundamental role in facilitating an individual’s academic and life successes. They are: executive function (EF) (Blair and Razza, 2007; Diamond and Lee, 2011; McClelland and Cameron, 2011; Moffitt et al., 2011; Monette et al., 2011; Karbach and Unger, 2014) and language abilities (Bashir et al., 1987; Bashir and Scavuzzo, 1992; Kastner et al., 2001; Clegg et al., 2005).

EF encompasses a wide range of cognitive abilities including, but not limited to, planning and goal setting, inhibition, working memory, attention, emotional control, shifting, and problem solving. The mental faculties of EF are particularly important during the early school years given that their neural basis is undergoing dynamic change and growth (Luciana and Nelson, 1998; Diamond, 2001; Zelazo et al., 2008; Karbach and Unger, 2014). Concomitant with evolving EF skills during early childhood is language development, which includes vocabulary, grammar, discourse, comprehension, and the focus of this study, speech articulation. Regarding articulation, speech development research has noted that in general, a number of speech sounds are not fully mastered in a child’s phonetic inventory until age 7 or beyond (Templin, 1957; Sander, 1972; Smit et al., 1990; Goldman and Fristoe, 2000). Speech articulation is a *skilled motor behavior* involving accurate placement and coordination of the oral effectors. Articulation errors are not uncommon in young children and may consist of phoneme (sound) substitution. For instance, in English, “s” and “sh” are contrasted phonemically; a young child may have difficulty differentiating the “s” and “sh” sounds, and as a result may produce the word “seep” as a substitute for “sheep” (Li et al., 2009).

A considerable body of empirical research and literature has documented an association between EF and language abilities in children. More specifically, language representation (Miller and Marcovitch, 2015), language fluency (Engelhardt et al., 2013), expressive and receptive language (Trainor, 2012; Petersen et al., 2015), and gesture use (O’Neill and Miller, 2013; Kuhn et al., 2014), have all been reported to be associated with EF. Trainor (2012) found that preschool children who demonstrated stronger language skills (receptive, expressive, content, and structure) had better EF as measured by the Behavior Rating Inventory of Executive Function—Preschool version (BRIEF-P), a multidimensional rating inventory that evaluates and assesses for EF impairment of preschool children in everyday activities.

It is evident that there is a connection between EF and language. What is less clear, however, is the nature of this association. Vygotsky, a pioneer in developmental psychology, was one of the first to posit that speech plays an essential role in a child’s development of particular cognitive skills such as planning, problem-solving, self-direction, and self-control (all within the realm of EF) (Vygotsky, 1997). To this day, many scholars assert that language is a factor underlying EF development (Barkley, 1997; Fatzer and Roebers, 2012; Landry et al., 2012; Kuhn et al., 2014; Botting et al., 2017). This notion is encapsulated by Wilbourn et al. (2012) such that cognitive processes that underlie goal-directed behavior are predicated on children’s use of language to regulate thoughts and behaviors.

Two longitudinal studies have provided support for the hypothesis that children’s language abilities may facilitate their EF. Kuhn et al. (2014) found that toddler’s gesture use at 15 months of age (e.g., pointing) predicted vocal development and social communication skills at 2–3 years of age, which in turn predicted EF abilities at 4 years of age. Petersen et al. (2015) found that receptive and expressive language abilities as a whole, predicted later self-regulation in children between the ages of 2 and 3. Self-regulation emerges early on in a child’s life (Kochanska et al., 2001) and involves different EF domains, such as inhibitory control, to promote goal-directed behavior (McClelland et al., 2007).

Although researchers have contended that language development is foundational in the emergence of EF, an alternative and equally plausible hypothesis is that language abilities are contingent on EF processes. It can be postulated that EF skills such as working memory, attention, inhibition, and planning are required to string together fluent and semantically correct sentences in a speech stream. Working memory is involved in the online storage and processing of verbal tasks. Children with specific language impairment (SLI) have been shown to have reduced capacity in working memory even after controlling for general language abilities (Archibald and Gathercole, 2006). This has also been evidenced in an fMRI study conducted by Tkach et al. (2011). When compared to controls, adolescents with speech sound disorders (SSD) showed hypoactivation in brain regions responsible for phonological memory while engaging in a non-word repetition task. In a study of typically developing 6-year-olds, Hayiou-Thomas et al. (2004) administered a linguistic task that attempted to mimic SLI by increasing task demands. The experimental design simulated both reduced memory capacity and reduced processing speed. They found that children with typical language development performed similarly to those with an SLI profile, suggesting that working memory disturbances can play a causal role in reducing linguistic performance.

Not only has working memory been shown to be implicated in language skills, but other EFs have also been reported. On an auditory attention task, Murphy et al. (2014) found that children with SSD had higher incidences of false alarms than controls and attributed this to impaired inhibitory control and/or selective attention (Murphy et al., 2014). Dodd and McIntosh (2008) found that preschool children with speech deficits performed more poorly than children with typical speech development on rule derivation and cognitive flexibility (higher order cognitive abilities that fall into the realm of EF). In another study, Engelhardt et al. (2010) examined speech production disfluencies in a population of ADHD individuals as this disorder is linked to impaired inhibitory control and response suppression. The authors found that the combined type of ADHD produced more repair disfluencies than controls, suggesting that impaired inhibitory control is implicated in speech errors (Engelhardt et al., 2010). These studies highlight the important contribution of EF in language production.

As we can see, there have been numerous reports evidencing a relation between language and EF abilities, but to our knowledge only one study has examined speech articulation proficiency in relation to EF. In a study investigating EF and phonological development in 4- to 5-year-olds, Eaton and Ratner (2016) aimed to address whether EF contributes to adult-like speech production abilities. Children with SSD and children with low-average and high-average speech skills took part in a battery of assessments. The battery consisted of eight performance based EF tasks (examining working memory, inhibitory control, and cognitive flexibility), spontaneous language production, and a picture naming task. Spontaneous language production was effectuated by a ten minute play session to examine for syntactic complexity and vocabulary diversity. The picture naming task assessed consonant accuracy in initial, medial, and final-word position by means of transcription methods. Results revealed that only one EF task was associated with speech sound accuracy. That is, children who performed better on the forward digit span task had stronger speech sound production. This task uses number sequencing to measure short term auditory memory. The authors propose that children who produce speech errors may have lower working memory capacity.

Accurate articulation is an aspect of language that requires intricate motor execution. Accordingly, a growing body of research has suggested a link between language development and motor behavior (Bates et al., 1986; Ramus et al., 2003; Esseily et al., 2011; Gonzalez et al., 2014a; Nelson et al., 2014). More specifically, stronger language abilities have been associated with a right-hand preference in infants (Bates et al., 1986; Esseily et al., 2011; Nelson et al., 2014) and in children (Gonzalez et al., 2014a). Specific to speech articulation, Gonzalez et al. (2014a) demonstrated that in typically developing 4- to 5-year-olds, the greater the right-hand preference for picking up small food items, the greater the differentiation between “s” and “sh” sounds. One reasonable interpretation to this finding is that both speech and precision grasping require fine motor control, and the right hand has been shown to be more adroit at grasping (Flindall et al., 2014).

These findings and others (Nip et al., 2011; Gooch et al., 2014) have suggested developmental parallels in EF, language, and motor control. Nip et al. (2011) conducted a longitudinal study on infants as they matured from 9 to 21 months. Developmental data examining cognition, language, and speech motor control was collected. Results revealed that communication was significantly correlated with articulatory kinematics. Interestingly, jaw speeds and all kinematics of the lower lip, including range of movement, were significantly correlated with attention and memory. These results suggest that the development between EF processes, articulatory refinement, and linguistic abilities may be intimately linked.

In view of the aforementioned evidence, the present study explored whether speech articulation, a fundamental component of language, is associated with EF. Unlike previous studies, we used a very precise acoustic analysis to examine how the degree of “s” and “sh” sound distinction is related to EF as measured by parent ratings in 4- to 6-year-olds.

We chose the “s” and “sh” distinction as a parameter of speech competency because this distinction has served as a valuable articulatory measure in phonetic research (Nittrouer, 1995; Perkell et al., 2004; Kraljic and Samuel, 2007; Li et al., 2009; Haley et al., 2010; Li, 2012; Holliday et al., 2015). Accurate production of the “s” and “sh” sounds (voiceless sibilant fricatives) involves complex motor behavior of tongue tip positioning. The main articulatory difference between the two fricatives is that the “sh” is produced with the tongue raised high in the mouth while positioning itself in a more posterior position than the “s” sound (Ladefoged and Maddieson, 1996). In gauging the articulatory accuracy of these gestures, phoneticians often apply an acoustic method of analysis known as spectral moment analysis. This statistical procedure consists of computing the mean frequency of the spectral distribution from the audio waveform, and is a property common to both “s” and “sh.” The clear acoustic separation in phonetic space between the two sibilant fricatives makes the centroid frequency a robust measure when investigating speech sound precision.

Research has indicated that young children have yet to reach mature production pattern status in their “s” and “sh” articulation as the distinction between the two sounds has been shown to be greater in adults than children (Nittrouer, 1995; Nissen and Fox, 2005). The degree of word-initial “s–sh” separation in children has been reported to increase with age: in 2- to 5-year-olds (Li, 2012; Holliday et al., 2015) and in 3- to 7-year-olds (Nittrouer et al., 1989). Although other sound contrasts follow a similar developmental trajectory (e.g., “r” and “l”), their acoustic mapping is not as clear-cut, and may deviate into similar acoustic space (Dalston, 1974; Espy-Wilson, 1992; McGowan et al., 2004). Therefore, the age-related progression noted in the “s” and “sh” distinction makes it a suitable developmental speech marker.

Preschool- and early school-aged children are experiencing rapid development in both motor abilities and EF (Livesey et al., 2006; Pennequin et al., 2010), with speech sound precision still on the brink of development, particularly with respect to the voiceless sibilant fricatives (Nittrouer, 1995; Li, 2012; Holliday et al., 2015). Sander (1972) proposes that maximal distinction of this fricative sound contrast is usually acquired by age 7. This makes 4- to 6-year-old children good candidates for the present study. In addition, a number of empirical research studies outlined above (e.g., Wassenberg et al., 2005; Dodd and McIntosh, 2008; Gonzalez et al., 2014a,b) investigated children that fell within the same age range, thus enhancing the ease of cross-study comparisons.

Our study examined whether speech articulation (a language skill requiring precise manipulation of the oral effectors), would reveal an association with a child’s EF skills. Furthermore, we aimed to determine whether articulatory abilities could serve as a predictor for the Global Executive Composite (GEC) score of the Behavior Rating Inventory of Executive Function (BRIEF) and also whether the GEC of the BRIEF could serve as a predictor for articulatory abilities. The rationale for this approach was motivated by empirical research that has suggested that EF may be predicated on language skills (e.g., Kuhn et al., 2014; Petersen et al., 2015) and that language may be contingent on EF processes (e.g., Hayiou-Thomas et al., 2004; Murphy et al., 2014). Our objective was to gain a more comprehensive understanding of the development of these processes so as to help guide future research, assessment, and early intervention strategies. If in fact speech articulation is related to EF, and even more so if it is shown to be a predictor of EF, children with speech difficulties could be at higher risk for EF deficits. Given the link between EF skills and both language and motor behavior, we hypothesized that more accurate speech articulation in children would be linked to stronger EF ratings.

## Materials and Methods

### Participants

Thirty-three children between the ages of 4 and 6 years old (15 female, 18 male) participated in the study (nine 4-year-olds—*M* = 4.69, *SD* = ±0.18; fifteen 5-year-olds—*M* = 5.36, *SD* = ±0.29; nine 6-year-olds—*M* = 6.33, *SD* = ±0.15). Participants were recruited via the posting of notices throughout the University campus, the University Daycare Center, and the Holy Spirit school division. Participants were naïve to the purpose of the study. This study was reviewed and approved by the University of Lethbridge Human Ethics Committee (Protocol # 2013-073) and caregivers provided written informed consent on their child’s behalf. All children were reported to be typically developing with no known behavioral, learning, or hearing impairments. Each child received a $10 gift card and a small toy for study participation.

### Procedure

#### Parent Questionnaires

After informed consent was obtained, caregivers completed two questionnaires. First, a general questionnaire (devised in-house) provided information about the child’s development in terms of whether they had ever been diagnosed with any sensory, motor, learning, hearing, cognitive impairments, or neurological conditions. Only those children free of any of these diagnoses were included in the study. Second, a BRIEF questionnaire assessed children’s EF of everyday behaviors (Gioia et al., 2000, 2003). The BRIEF is commonly used in research and clinical practice to assess for EF problems and has been attested as an ecologically valid and reliable measure of EF (see Donders, 2002 for a review). The BRIEF questionnaire asks guardians to rate behaviors exhibited by their child within the last 6 months as occurring often, sometimes, or never. The standard version of the BRIEF is an 86-item questionnaire and the preschool version (BRIEF-P) is comprised of 63 questions. The standard version of the BRIEF was completed by guardians of children who were 5–6 years of age while the BRIEF-P, was answered by guardians of children who were 4 years of age. Both versions share five common EF subscales: inhibit (control impulse), working memory (store and manipulate necessary information in order to complete a task at hand), shift (flexibly modify behaviors according to situational demands), emotional control (modulate emotional behavior accordingly), and plan/organize (set goals and develop steps). Each subscale yields a raw response score that is then converted into a corresponding *t*-score based on age and gender. These subscales, as well as a GEC score (the child’s all-encompassing score of EF) were used as the variables in the study.

#### Speech Task

In order to examine children’s speech articulation, a word-repetition task was administered. Specifically, “s” and “sh” ([s] and [ʃ], respectively in the International Phonetic Alphabet) were targeted in word-initial position and the accuracy of the children’s speech production was assessed. Participants were tested individually in a quiet room where they were seated at a desk in front of a computer monitor. The computer program *Show* & *Play* (Edwards and Beckman, 2008) was used to couple auditory stimuli with corresponding visual stimuli. Participants were instructed to listen to the computer play a word through the speakers (Logitech Z205, model: S-00094) while watching the computer screen display a matching picture. They were asked to repeat the word back into the microphone after it was done playing. After the child articulated the word into the microphone, the experimenter would click a computer mouse to proceed to the next stimulus. With the experimenter in control of each individual trial, the child was able to proceed at their own pace so that processing speed was not a confound. Non-automaticity of the computer program also allowed the experimenter to replay a word if a child missed the cue or was not paying attention. A Shure SM87A microphone was used, with a sampling rate of 44,100 Hz and was placed at distance of approximately 15 cm from the participant’s mouth. Children’s speech production was recorded using a Marantz flashcard recorder (model: PMD661).

#### Stimuli

The experimental stimuli consisted of 18 target words with fricatives ([s], [ʃ]) in word-initial position, preceded by three consistent vowel environments: “i,” “a,” “u” ([I], [ae], [Λ]) (see **Table 1**). In addition to the experimental words, 35 non-target filler words were inserted with the test items. All participants produced nine tokens for each target sound. Three randomized word lists were generated and administered to participants in a counterbalanced fashion. A practice trial of five tokens was administered prior to the experimental condition. The practice words were independent from the testing stimuli and provided children with an opportunity to feel comfortable with the experimental design. Audio prompts consisted of natural pre-recorded speech from a native female English speaker.

**Table 1:** Stimuli list.

Target sound	Vowel			
s	I	Sick	Sit	Silly
	ae	Salad	Salmon	Sandwich
	/∖	Sun	Sucker	Subway
sh	I	Ship	Shin	Shiver
	ae	Shadow	Shallow	Shack
	/∖	Shuffle	Shut	Shovel

## Results

### Descriptive Statistics

#### Behavior Rating Inventory of Executive Function

Behavior Rating Inventory of Executive Function scores were calculated following the scoring procedure provided in the BRIEF and BRIEF-P manuals (together referred as BRIEF assessments henceforth; Gioia et al., 2000, 2003). A raw score for each subscale was obtained and then converted into a *t*-score, where lower *t*-scores are associated with better EF. Given that the *t*-score provides an indication of how a child scores relative to children in the standardization sample, it was the *t*-scores that were used for statistical analysis. The BRIEF assessments are separated into EF subscales (BRIEF = 8 subscales, BRIEF-P = 5 subscales). The subscales shared across both questionnaires were examined (inhibit, shift, emotional control, working memory, and plan/organize) as well as the global EF test score (GEC). BRIEF information from two children was incomplete leaving a total of 31 cases for analysis. Mean *t*-scores and standard deviation values for each age group and all six domains as rated by parents on the BRIEF assessments are displayed in **Table 2**. In order to examine how the *t*-scores of the overlapping scales shared across the two versions of the questionnaire compare, an independent sample *t*-test was performed. Questionnaire version (BRIEF vs. BRIEF-P) was inserted as the grouping variable while the test variables consisted of *t*-scores for: inhibit, shift, emotional control, working memory, and plan/organize, and GEC. No significant differences in *t*-scores were found between the two versions of EF rating. Given these findings, the subscales and GEC shared across both BRIEF assessments were collapsed together.

**Table 2:** Mean and standard deviations for each BRIEF scale separated by age group.

	GEC	Emotional control	Working memory	Plan/organize	Shift	Inhibit
Age	Mean ±*SD*	Mean ±*SD*	Mean ±*SD*	Mean ±*SD*	Mean ±*SD*	Mean ±*SD*
4 (*n* = 8)	50.0 ± 11.5	50.0 ± 11.3	52.1 ± 10.8	46.5 ± 8.3	48.1 ± 5.3	51.5 ± 12.7
5 (*n* = 15)	47.8 ± 7.2	49.4 ± 7.1	47.9 ± 9.0	47.0 ± 4.9	52.1 ± 10.2	48.5 ± 8.1
6 (*n* = 8)	56.9 ± 9.3	58.1 ± 13.1	59.6 ± 6.8	55.6 ± 7.5	51.6 ± 14.6	54.3 ± 7.1

#### Speech Analysis

Each child’s speech recording was manually segmented into individual words using the speech analysis software Praat Version 5.3.3.9 (Boersma and Weenink, 2013). A fine-grained analysis of the fricative segments ([ʃ], [s]) was conducted to extract the acoustic spectral centroid frequency, defined as the weighted mean frequency of the sound noise spectrum (Forrest et al., 1988; Jongman et al., 2000). The spectral centroid frequency is a quantifiable acoustic variable that can be subjected to statistical analysis, with the “s” and “sh” contrast serving as a marker in the development of sound acquisition. The first step to extracting the spectral centroid frequency involved manually labeling all segments of “s” and “sh” using Praat software. The beginning as well as the end of the acoustic energy for each individual segment was marked. Segments were then processed through the Multitaper Package (Rahim, 2010) in R (R Development Core Team, 2011). A 40 ms spectrum slice surrounding the middle of each “s” and “sh” sound segment was made, from which values for the spectral centroid frequency of [s] and [ʃ] were calculated. An accurately produced [s] is expected to produce a significantly larger spectral centroid frequency value than [ʃ]. By calculating the difference between the values of [s] and [ʃ] for each individual child, it is possible to obtain a clear measurement of the acoustic differentiation of the two phonetic segments. Greater differentiation indicates that a speaker is producing each sound in a distinctive manner, creating a contrast between the two fricatives. Mean centroid frequency and standard deviation values for both fricatives in each age group are found in **Table 3**. In addition to spectral centroid frequency extraction, phoneme transcription was conducted as a supplementary analysis for descriptive statistic purposes. This method consists of sounds being manually transcribed by trained researchers in order to judge the accuracy of a sound segment (e.g., Shuster and Wambaugh, 2000; Preston and Edwards, 2010). All word-initial sounds (18 per participant) were transcribed by two native English speaking research assistants. Each targeted sound was judged and labeled as either a correct or incorrect pronunciation. The total number of speech errors for all word-initial targeted sounds were recorded for each child and for each sound. A total of 53 manually transcribed speech errors were noted for a total 9.5% articulation error rate. 8.4% of these errors were for [ʃ] and 1.0% for [s]. Contribution to the error rate by age category is as follows: 3.6% for the 4-year-olds, 3.9% for the 5-year-olds, and 2.0% for the 6-year-olds.

**Table 3:** Mean centroid frequency for each fricative and mean centroid frequency difference between fricatives separated by age group.

	[s]	[ʃ]	[s]–[ʃ]
Age	Mean ±*SD*	Mean ±*SD*	Mean ±*SD*
4 (*n* = 8)	7595.2 ± 1084.5	5318.4 ± 929.7	2276.8 ± 1642.4
5 (*n* = 15)	7277.4 ± 836.4	5072.7 ± 997.7	2204.7 ± 1091.6
6 (*n* = 8)	7483.5 ± 948.9	4944.6 ± 1092.0	2538.9 ± 1233.0

### Correlation Analyses

The objective of this study was to determine whether the five subscale scores obtained on the BRIEF assessments (inhibit, shift, emotional control, working memory, plan/organize) in addition to the overarching summary score (GEC) would correlate with the mean centroid frequency differences between [s] and [ʃ] (measured in hertz). To correct for multiple comparisons in the correlation analysis, the Benjamini and Hochberg (1995) procedure was applied with a false discovery rate (FDR) of 5%. This method has shown to be powerful in detecting true associations while correcting for the expected proportion of false positives. Each individual *p*-value was compared to its Benjamini–Hochberg critical value to determine corrected significance thresholds.

**FIGURE 1:**
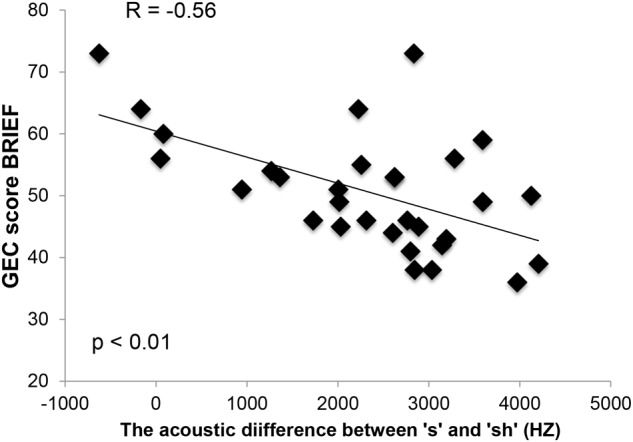
The graph depicts the relation between the GEC score on the BRIEF inventories (standard score) and the acoustic difference between “s” and “sh.”

Results showed that there were significant negative correlations between [s] and [ʃ] acoustic differentiation and all scales of the BRIEF, including the GEC. All correlations remained significant after applying FDR. Results are as follows: GEC (*r* = -0.56; *p* = 0.001); Inhibit (*r* = -0.50; *p* = 0.002); Shift (*r* = -0.38; *p* = 0.018); Emotional Control (*r* = -0.44; *p* = 0.007); Working Memory (*r* = -0.42; *p* = 0.009); Plan/Organize (*r* = -0.40; *p* = 0.012). **Table 4** provides the FDR adjusted *p*-values. Effect sizes ranged from medium to large with the lowest being observed in the Shift subscale (*r* = -0.38) and the largest being observed in the GEC (*r* = -0.56). As shown in **Figure 1**, the overall BRIEF global composite score is negatively correlated with the acoustic separation between the “s” and “sh” sounds. In other words, better GEC scores (lower scores) are highly correlated with stronger articulatory abilities in distinguishing the [s]–[ʃ] contrast.

**Table 4:** Correlation of BRIEF scores in relation to the acoustic difference between “s” and “sh.”

Variables	*R*	*P*-value	FDR adjusted *P*-value
GEC	–0.56	0.001	0.008
Inhibit	–0.50	0.002	0.016
Shift	–0.38	0.018	0.050
Emotional control	–0.44	0.007	0.025
Working memory	–0.42	0.009	0.033
Plan/organize	–0.40	0.012	0.042

Although BRIEF *t*-scores are standardized, thus adjusting for across age cohort comparison, chronological age in days was entered into the correlation matrix as it was of interest to examine age with respect to the number of transcribed speech errors and the acoustic differentiation between [s] and [ʃ]. BRIEF subscales and the GEC were not correlated with age, with the exception of one subscale, Plan/Organize (*r* = 0.445; *p* = 0.012). Results did not reveal a statistically significant correlation between age and [s]–[ʃ] differentiation (*r* = 0.18; *p* = 0.335), or between age and total number of speech errors (*r* = -0.16; *p* = 0.379). As might be expected, there was a significant correlation between the [s]–[ʃ] differentiation and transcribed speech errors (*r* = -0.79; *p* < 0.001).

### Regression

Two simple OLS regression analyses were conducted. The first analysis examined whether the GEC could serve as a predictor for [s]–[ʃ] differentiation [*F*(1,29) = 12.9; *p* = 0.001, *R*^2^ = 0.284] and indicated that the GEC accounts for approximately 28.4% of the variance in [s]–[ʃ] differentiation; however, adding age to the regression model [*F*(2,28) = 10.6; *p* < 0.001, *R*^2^ = 0.389] significantly increased the proportion of the variance in [s]–[ʃ] differentiation, and accounted for 38.9%. Subsequent moderation analysis did not reach significance (*p* = 0.069). **Table 5** displays the regression coefficients for GEC and age. The unstandardized coefficient shows that with every unit of increase in GEC, the [s]–[ʃ] differentiation decreases by approximately 73-fold. In other words, higher scores on the GEC (lower abilities) leads to less proficient differentiation in [s]–[ʃ] speech sound production. After controlling for age, the effect of GEC on [s]–[ʃ] differentiation increased by about 10-fold.

**Table 5:** Results of the first regression analysis for the GEC score and chronological age in days in relation to the dependent variable of acoustic difference between [s]–[ʃ].

		Unstandardized coefficients		Standardized coefficients	
		*B*	Standard error	β	*t*
Model 1	GEC	–73.18^∗∗^	20.37	–0.56	–3.59
Model 2	GEC	–83.93^∗∗∗^	19.68	–0.63	–4.27
	Age in days	1.75^∗^	0.78	0.33	2.24

*Significant at ^∗^*p* < 0.05; ^∗∗^*p* < 0.005; ^∗∗∗^*p* < 0.001.*

The second analysis examined whether [s]–[ʃ] differentiation could serve as a predictor for GEC. Results were the same as the above analysis with the exception of the moderation analysis (*p* = 0.061) and the regression coefficients (see **Table 6**). The unstandardized coefficient shows that with every unit of increase in [s]–[ʃ] differentiation results in a decrease of 4 in the GEC score (when measured in kilohertz).

**Table 6:** Results of the second regression analysis for the acoustic difference between [s]–[ʃ] and chronological age in days in relation to the dependent variable of the GEC score.

		Unstandardized coefficients		Stan dardized coefficients	
		*B*	Standard error	β	*t*
Model 1	[s]–[ʃ]	–0.004^∗∗^	0.001	–0.56	–3.59
Model 2	[s]–[ʃ]	–0.005^∗∗∗^	0.001	–0.62	–4.27
	Age in days	0.014^∗^	0.006	0.36	2.44

*Significant at ^∗^p < 0.05; ^∗∗^p < 0.005; ^∗∗∗^p < 0.001*.

## Discussion

Many previous investigations documenting the interrelatedness between language and EF abilities have placed particular emphasis on higher order aspects of language (e.g., fluency, receptive, and expressive abilities, language representation, and sentence comprehension) (Montgomery, 1995; Trainor, 2012; Engelhardt et al., 2013; Miller and Marcovitch, 2015), or have relied on transcription methods to measure speech accuracy in relation to cognitive performance-based EF measures (Eaton and Ratner, 2016). These studies have suggested that more proficient language abilities are associated with stronger EF skills. In addition, previous studies have highlighted a connection between motor behavior and EF (Sergeant, 2000; Wassenberg et al., 2005; Livesey et al., 2006; Gonzalez et al., 2014b). Given that speech production is both an aspect of language and motor skills, the unique contribution of the present examination was the focus on speech articulation as measured by a very precise acoustic analysis and its association with EF as measured by parent behavioral ratings. To this end, children aged 4–6 took part in a speech production task targeting word-initial [s] and [ʃ] while their parents filled out the appropriate BRIEF assessment as a measure of their child’s EF. The acoustic difference between “s” and “sh” was the measure used to determine articulatory ability. We hypothesized that stronger speech articulation abilities would reveal better EF scores in children.

The results of the present study showed that the more accurate a child’s speech articulation, the better (lower) their overall score on the BRIEF inventories. Thus, participants who exhibited a higher degree of acoustic separation between their “s” and “sh” sound production in a word-repetition task, obtained better scores for the GEC and for *all* five subscales of the BRIEF assessments (inhibit, shift, emotional control, working memory, plan/organize). Furthermore, regression analyses demonstrated that the all-encompassing GEC score was a significant predictor of [s]–[ʃ] differentiation and that [s]–[ʃ] differentiation was a significant predictor of GEC. After examining the regression coefficients, it appears that the more robust regression analysis was the former, that is, GEC has a greater influence on [s]–[ʃ] differentiation than the other way around. When age was included as a covariate, there was a significant increase in the proportion of variance in the dependent variable in both regression analyses. However, there was no interaction. This finding indicates that age is not a confounder, but is certainly a covariate that is influencing the effect of the GEC on [s]–[ʃ] differentiation and vice versa. To our surprise, results did not reveal a statistically significant correlation between age and [s]–[ʃ] differentiation or between age and total number of speech errors. Further inspection into the descriptive statistics of the transcription error rates revealed that this is likely explained by the 4- and 5-year-olds who showed very similar speech articulation patterns.

Although the cross-sectional design of the present study cannot be conclusive of causality or directionality in the relation between speech articulation and EF, the significant results of both regression analyses suggest that this association may be reciprocal. The exact nature of this relationship needs to be further explored and validated by implementing a longitudinal study design. The more robust regression analysis of the two, where the GEC of the BRIEF assessments served as a strong predictor for [s]–[ʃ] differentiation, points to the interpretation that speech production is contingent upon EF processes. Likely, many faculties of EF are pertinent in conceptualizing and formulating language in real-time. Conceptualization and formulation are two of three processes referred to in the theory of lexical access in speech production (Levelt et al., 1999). In order to communicate effectively, a speaker must first bring to mind a desired concept and string together the intended message, followed by second, the correct encoding system adopted by that language. The third and last stage involved in the transmission of speech, is articulation.

Although early theoretical frameworks and speech models have alluded to the involvement of certain EFs such as planning, monitoring, and inhibition in speech output (e.g., Laver, 1980; Dell, 1986; Levelt, 1989), EF as a more comprehensive construct has yet to be effectively delineated into speech production models. Speech articulation involves harmonious communication between the language and motor domains (Smith and Goffman, 2004) that is likely to be contingent on many EF processes. Florez (2011) asserts that self-regulation is involved in the necessary responses carried out by the motor and language systems. Self-regulation draws from inhibition and emotional control, two EF domains that revealed a significant correlation with respect to [s]–[ʃ] differentiation in the present study. In addition, the three other EF domains (shift, plan/organize, working memory) shared across the BRIEF assessments, revealed a similar effect. It is reasonable to speculate that the remarkable coordination and complexity involved in the execution of forming a pronounced contrast between fricatives (s-sh), incorporates the use of all five EFs investigated. Shifting is likely to be involved in producing accurate speech segments by drawing on rule derivation and cognitive flexibility. Specific to the present experiment, the child must be able to shift their articulatory output between the [s] and [ʃ] contrast to produce each speech sound in a distinctive manner. Next, the ability to plan and organize would involve stringing together the intended and appropriate speech utterance, and is also associated with motor behavior (Gonzalez et al., 2014a). Lastly, working memory is involved in the online verbal storage system needed to update phonological rules, and has shown to be implicated in speech production as reported by Eaton and Ratner (2016).

Articulation is an aspect of language that requires intricate motor execution. Interestingly, children with SLI may exhibit fine and gross motor skill deficits (Hill, 2001; Zelaznik and Goffman, 2010), have lower EF on the BRIEF-P (Wittke et al., 2013) and lower scores on direct performance based measures of EF (Roello et al., 2015). An integrated approach highlighted by Sharma and Cockerill (2014), proposes that a range of developmental processes interact to form a *dynamic system* where speech, language, motor, and EF components (among others) develop in a cohesive manner. It has been proposed that EF begins to emerge in the first year of life (Diamond, 2006), as does vocal development (Guenther, 1994), and motor skills (Flensborg-Madsen and Mortensen, 2017; Gonzalez and Sacrey, 2018). Functional imaging reports provide evidence for the interrelatedness between EF, language, and motor systems. The neural networks that support EF processes are numerous and complex—the prefrontal cortex acts as a mediator and relies on connections with virtually all brain regions including motor, premotor, temporal, and parietal areas (Stuss and Benson, 1984; Kolb et al., 2016). Similarly, the frontal and temporoparietal cortices are recognized to play a critical role in language function (Ojemann, 1991). Accordingly, Fuster (2002) highlights the importance of lateral prefrontal cortex development to serve both EF and language. Yet, another brain region that has been shown to be active in both EF and language, particularly speech, is the cerebellum (see Ackermann et al., 2007 for a review). For instance, children with cerebellar mutism followed by dysarthria (slurred speech) also present with impaired EF (Riva and Giorgi, 2000). Together, these studies suggest the interesting possibility that the neural networks supporting speech production are also responsible for emergent EF during early childhood.

If common neurobiological pathways are responsible for the development of these abilities, then an impingement on brain regions implicated in these functions could lead to comorbidity (as outlined in Bishop et al., 2014).

The findings of our study suggest that children with articulation difficulties may be at higher risk for executive dysfunction. Furthermore, motor impairments might also coexist (Gooch et al., 2014). The implications of the comorbidity across these domains are critical for assessment and intervention purposes where more broad-based approaches will prove useful. Practitioners such as speech language pathologists and occupational therapists may want to consider an integrated method of assessment to expand their test battery to include measures of EF. It will also be of essence to provide referrals to one another when comorbidity is suspected.

Intervention studies have shown that treatment plans targeting non-linguistic cognitive processes may lead to improvement in language abilities in individuals with aphasia (Kohnert, 2004; Coelho, 2005) and SLI (Ebert and Kohnert, 2009). Ebert and Kohnert (2009) found that brief but intensive intervention targeting non-linguistic cognitive processing skills over a 4-week period led to enhancement in certain language abilities in two school-aged children with SLI. Expressive language and sentence formulation were two areas that were most positively impacted by the treatment. These findings lend support to the more powerful regression analysis in the current study where EF predicted speech articulation. The results of these studies are promising in that cognitive interventions could potentially lead to an amelioration in language skills. Further research is needed to investigate this possibility.

Our study provides evidence for an integrated system in the development of EF and speech abilities, yet it is important to address the limitations of this experiment. First, it is not possible to dissociate the motor aspect of speech articulation from the broader language system. This makes it difficult to tease out the ultimate driving force behind the findings. Future work in this area may wish to dissect these factors by including higher order language assessments and motor batteries, in addition to speech articulation and EF measures. But that said, it is likely that the results speak to the combination of language, motor, and EF as a whole.

Of course, the cross-sectional design of this study does not allow for causal inferences to be drawn. Longitudinal study designs will be needed to determine any causal relationship between EF and speech articulation. Future studies may wish to collect data from toddlerhood through to middle childhood to encompass a wide developmental range. Findings of such studies may help to guide theoretical frameworks integrating the role of EF in speech production.

Because of some missing demographic information such as socioeconomic status (SES) and parental education, the findings of this study need to be interpreted with some caution. Because this information was not available, we cannot draw any conclusions of their potential impact. However, it can be noted that SES accounts for less than 2% of the variance in EF rating scores in the BRIEF-P and at most 5% of the variance in the BRIEF, while parental education may account for 5% of the variance at most in both BRIEF assessments (Gioia et al., 2000, 2003).

Another limitation to this study is the small sample size. However, the results of medium to large associations among BRIEF scale scores and [s]–[ʃ] differentiation in addition to the large regression coefficient results, reinforce the validity of our findings. When the analysis was focused on the 4- and 6-year-old children, resulting in a much smaller sample, the regression coefficients remained almost unchanged (data not shown). This observation lends credibility to our results.

Lastly, EF was measured via parent ratings provided by BRIEF assessments. Although this standardized inventory has been attested in identifying EF impairments, performance-based EF tasks may tap into different EF faculties than behavioral ratings (Anderson, 2002; Mahone et al., 2002; Vriezen and Pigott, 2002; Mahone and Hoffman, 2007). Mahone and Hoffman (2007) found consistently low correlations between the BRIEF-P and performance-based EF measures. Inventories such as the BRIEF provide an account of rational goal pursuit behavior in everyday settings whereas cognitive performance-based tasks capture efficiency of performance in an optimal setting (Toplak et al., 2013). Having said that, it has been suggested that the BRIEF assessments are more sensitive in detecting everyday EF deficits than in-lab measures. That is to say, performance-based tasks are administered in a structured, novel, quiet, and one-on-one testing environment that is not representative of everyday life (Sherman and Brooks, 2010).

In sum, the results of the present investigation demonstrate the imbricated nature of EF and speech articulation accuracy. Our findings suggest that the elaborative system of speech production requires the use of higher order EF skills and that speech production may also serve as predictor for EF. Preschool and school-aged children who were rated by their parents to have better overall EF, showed stronger speech articulation proficiency as measured by a very precise acoustic analysis. Our study provides novel evidence for the interrelatedness between many domains of EF and an integral component of language: speech sound production.

## Author Contributions

NN conducted literature search, created stimuli list, implemented experimental set-up, recruited participants, collected data, scored and coded the data, conducted statistical and acoustic analysis, wrote the manuscript draft, and finalized all revisions. CG contributed to conceptualizing the research design, supervised data collection, participated in bi-weekly research meetings, helped in conducting data analysis, in interpreting the results, and with writing and editing the manuscript. FL contributed to creating the research design and refining research ideas, conducted acoustic analysis, and helped in interpreting the results and editing the manuscript. RG helped to refine research ideas, participated in bi-weekly research meetings, helped with statistical analysis, in interpreting the results, and with writing and editing the manuscript.

## Conflict of Interest Statement

The authors declare that the research was conducted in the absence of any commercial or financial relationships that could be construed as a potential conflict of interest.
